# Doping in elite cycling: a qualitative study of the underlying situations of vulnerability

**DOI:** 10.3389/fspor.2024.1482103

**Published:** 2024-11-22

**Authors:** Valentine Filleul, Fabienne d'Arripe-Longueville, David Pavot, Hugo Bimes, Jacky Maillot, Eric Meinadier, Denis Hauw, Karine Corrion

**Affiliations:** ^1^ LAMHESS, Université Côte D’Azur, Nice, France; ^2^ Department of Marketing, Business School, Université de Sherbrooke, Sherbrooke, QC, Canada; ^3^ Medical Department, French Federation of Cycling, Paris, France; ^4^ Faculté des Sciences Sociales et Politiques, Institut des Sciences du Sport, Université de Lausanne, UNIL, Lausanne, Switzerland

**Keywords:** doping vulnerabilities, maladaptive motivation, cycling, performance-enhancing drugs, interviews

## Abstract

Doping is considered a critical deviant behavior in competitive sports, and particularly in cycling, even though the phenomenon remains limited in sports in general. Previous qualitative studies have contributed to identify situations of vulnerability to doping in athletes. However, much of the research tends to focus on singular dimensions of vulnerability, such as physical or psychological aspects. The present study aimed to extend existing knowledge by concurrently exploring and attempting to categorize different types of situations of vulnerability that predispose elite cyclists to engage in doping. Ten high-level French-speaking doped cyclists were recruited (*M*age = 49; *SD* = 14.63, two women). Semi-structured interviews were conducted. Both deductive and inductive thematic analyses were performed. Our results highlighted four types of vulnerability situations: (a) psychological (e.g., negative affects, maladaptive motivation, depression), (b) physical (e.g., exhaustion, impairments, injuries), (c) relational (e.g., organized doping, control, psychological or sexual harassment, social approval of doping), and (d) contextual (e.g., cycling culture, weather conditions, competitive stakes). By providing a clearer categorization of the situations of vulnerability that converge toward doping in sport, this comprehensive study allows for a holistic understanding of the various vulnerabilities. It paves the way for future research on related vulnerabilities and dispositional factors. Practically, it should also improve doping screening and prevention, and provide more favorable conditions for athletes.

## Introduction

The World Anti-Doping Agency (WADA) defines doping as: “The occurrence of one or several Anti-Doping Rule Violations (ADRVs)’ as stated in the World Anti-Doping Code (e.g., art. 2.1: the presence of a prohibited substance or its metabolites or markers in an athlete's bodily specimen (1). The consequences of doping are multifaceted, and among them health-related outcomes due to the consumption of performance-enhancing substances are particularly prominent (2). Athletes also expose themselves to sporting sanctions as well as severe psychosocial repercussions in the event of positive testing (3). Moreover, the ramifications of doping extend to those who do not engage in the practice, as evidenced by cyclists who suffer from the tarnished reputation of their sport. These athletes often feel compelled to engage in even more coercive processes to regain trust and credibility (4).

Elite athletes, broadly speaking, are subject to specific risks due to the intense pressure and expectations placed upon them (5). Cycling is an exceedingly demanding sport that requires command of a diverse set of skills and attributes such as endurance, strength, speed, technical proficiency, and tactical awareness [e.g., (6–8)], and it lies at the intersection of individual and team-based endeavors (9). The sport is widely covered by the media and highly professional, leading to heightened expectations on the part of fans, media, and sports managers alike (7, 10). Athletes in cycling are therefore subject to prolonged and intense efforts, significant risks, and multiple types of pressure. In addition, they often face job insecurity (11, 12). Performance enhancement and health preservation concerns among cyclists can be triggering factors for doping practices. Insufficient recovery time, recurring injuries, or nutritional deficiencies are challenges they often struggle to manage cleanly (13) and the financial pressure can, in some cases, encourage risky behaviors (12).

Meta-analyses and systematic reviews sought to consolidate the findings of numerous studies to develop a robust understanding of doping behavior, each grounded in distinct theoretical frameworks. For example, Backhouse et al. (14) provided a comprehensive view by integrating both the Theory of Planned Behavior (TPB) (15) and Achievement Goal Theory [AGT, (16, 17)], identifying key variables associated with vulnerability to doping in competitive athletes. Their findings highlighted determinants such as male gender, the consumption of dietary supplements, early specialization in sports, and the number of years in elite sports. Psychological factors, including low self-esteem, compromised integrity, and high levels of trait anxiety, were also recognized as contributing elements (14). In a more focused meta-analysis, Blank et al. (18), grounded specifically in TPB, investigated predictors of doping intention, susceptibility, and behavior in elite athletes. Their analysis identified situational temptation, attitudes, and subjective norms as primary predictors of doping, aligning with TPB's emphasis on individual perceptions and social influence in shaping behavior. Building on these psychological insights, Bandura's Social Cognitive Theory (19, 20) introduced the concept of self-regulatory efficacy, which plays a critical role in regulating behavior. Athletes with strong self-regulatory efficacy were more likely to engage in behaviors that brought them satisfaction and self-confidence while avoiding behaviors that could lead to self-condemnation (19, 20). Bandura's theory contributes to explain how athletes might bypass internal moral constraints through moral disengagement, allowing them to rationalize unethical behavior. This process might increase the risk of doping by dissociating athletes from the emotional self-sanctions associated with reprehensible behavior (21, 22).

Certain time periods have been identified as particularly critical to the emergence of doping behavior [e.g., (23)], such as adolescence (24), entry into the professional world [e.g., (12, 25, 26)], or the end of an athlete's career [e.g., (25)]. Beyond these periods inherent to an athletic career, specific situations are associated with vulnerability. Situations of vulnerability should be understood as periods of weakness during which an individual's integrity is, or can be, compromised, diminished, or altered (27). Overbye et al. (28) referred to these situations as “setback situations” and others as tipping points, or periods of personal distress (29, 30).

Through in-depth interviews with elite cyclists and analysis of their various discourses, situations of vulnerability that predispose athletes to doping have been identified [e.g., (25, 26, 31, 32)]. These athletes are generally highly committed to their sport, which requires a significant investment of resources. The need to mobilize both physical, mental and social resources often leads to substance use.

Some vulnerable situations seem to primarily be due to physical aspects. Testimonies of doped athletes indicate that the decision to dope often follows changes in their athletic performance, either in terms of declining or inconsistent results (11, 29, 33). Engagement in competitive sports is associated with experiences of psychological and physical discomfort, as posited by Hauw and Bilard (29). Furthermore, the structural organization of these activities often neglects to provide sufficient opportunities for psychological recovery, such as relaxation, diversion, and dual career planning, thereby exacerbating the issue (29). In the context of cyclists, this lack of space for mental recuperation can be particularly problematic given the intense physical and psychological demands of the sport. Several studies have observed that athletes began to use doping substances after particularly intense training sessions, or during post-injury periods [e.g., (13, 34, 35)], as a means of recovery (29, 36).

Other situations of vulnerability appear to be more psychological in nature. Athletes seem driven by a “win-at-all-costs” motivation that increases the risk of doping (36). They describe life experiences during which doping occurs as stressful and burdensome (29) and experience specific and fluctuating psychological states marked by feelings such as emptiness, anticipation, disappointment, or frustration (11). The use of performance-enhancing substances allows athletes to mitigate psychological stress that may not always be consciously recognized (37). It seems that athletes who are distressed and suffering are more likely to turn to doping as a coping mechanism to deal with their struggles [e.g., (11, 38)].

Other works have pointed to situations of relational vulnerability. For instance, peer pressure (39) or situations of control, characterized by unhealthy coach-athlete relationships, can have a detrimental, ambiguous, or incomprehensible impact on athletes, thereby increasing the risk of doping [e.g., (28, 30, 40)].

Circumstances such as team expectations and coaching pressure appear to act as control parameters for the decision to dope or not [e.g., (11, 25)]. These factors seem to intensify when the competitive and financial stakes are high, particularly for cyclists who must reconcile multiple professional commitments due to job insecurity (12), suggesting the presence of situations of contextual vulnerability.

### The present study

While a number of psychological factors that explain and determine doping in athletes have been identified and have gained scientific legitimacy, it is important to note that understanding of the phenomenon in sports remains limited (18, 41). Previous qualitative studies have made valuable contributions to the understanding of situations of vulnerability to doping in athletes [e.g., (11, 29, 37)]. However, much of the research tends to focus on singular dimensions of vulnerability, such as physical or psychological aspects. This fragmented approach may prevent researchers from gaining a comprehensive understanding of how multiple forms of vulnerability can converge toward doping behavior. Furthermore, attempts to categorize these different types of vulnerabilities are lacking, thus limiting the possibility of applying findings to screening, detection, and better prevention of doping. Therefore, the present study aimed to extend previous knowledge by simultaneously exploring and attempting to categorize different types of situations of vulnerability that predispose elite cyclists to engage in doping.

## Methods and materials

### Methodological underpinnings

A qualitative approach was used because it was particularly suited to the exploratory nature of the main research questions. This approach involves the investigation and the understanding of how individuals or groups attribute significance to social or human issues (42). We adopted an interpretivist lens, grounded in a relativist ontology and constructionist epistemology (43). This approach allowed us to better understand how athletes themselves perceive and experience these situations. Our epistemological stance was constructivist, meaning that it emphasized the importance of understanding how individuals make sense of their experiences and the social constructions that influence these meanings (44). This research paradigm aligned well with the complexities of understanding the nuanced and multifaceted nature of doping behavior, which is shaped not only by individual psychological factors but also by broader social and cultural factors. As researchers in the human and social field, we value research that is useful and helpful (45). In line with our values, we believe that this work will contribute to sporting integrity and athlete welfare.

Because the authors of this article are co-constructors of knowledge and interpret the meaning of the experiences shared during the interviews, some of their characteristics must be acknowledged. The researchers are all sport scientists, experts on doping questions, and have under-graduate, post-graduate or doctoral level degrees. FAL, KC, and DP have over 15 years’ experience conducting research in the sport psychology field and in anti-doping education. JM and EM are sport physicians and specialists of cycling. VF is completing a PhD and HB has a master's degree, and both have focused their studies on the question of doping in cycling. VF has been the antidoping consultant of a French sport federation for 4 years. VF, who led all the interviews, has been trained in conducting qualitative research and has already been involved in qualitative research in the past (46, 47).

### Participants and recruitment

The interviewees consisted of ten participants including two females, who were current (*n* = 3) or former cyclists (*n* = 7) recruited on a voluntary basis from different French-speaking regions with the help of national federations (French Cycling Federation, Quebec Cycling Federation) and partner Anti-Doping Organizations. The mean age of participants was 49.0 years (*SD* = 14.6).

The inclusion criteria were intentionally permissive to capture a broad range of experiences. There were no restrictions based on gender, age, or level of competition. The only prerequisites were that the athletes had to be native French speakers and report having intentionally consumed prohibited substances at least once during their sports career. To achieve qualitative saturation, the recruitment was expanded beyond national borders. Seven of the participants came from France, the other three from Quebec. Nine of them had achieved a professional career.

Initial email outreach to positively tested French cyclists yielded a low response and led to follow-up calls and snowball sampling (i.e., chain referral), a strategy used in the context of doping research [e.g., (48)]. Despite efforts, the sample size remained unsatisfactory (*n* = 7) and was slightly expanded (*n* = 3) by recruiting from other French-speaking regions through national cycling federations. Recruitment stopped after data saturation was reached, notably when the themes and patters that emerged from the last interviews were completely redundant with previous data (49). We also ensured that our sampling strategy was robust, and that a diverse range of participants with different perspectives and experiences had been included. Furthermore, we used a rigorous approach to systematically analyze the data, which allowed us to identify the most salient themes. These factors provide strong evidence that qualitative saturation was achieved, and that our findings are reliable.

### Ethical considerations

The procedure was approved by the Ethical Committee for Non-Interventional Research of Université Côte d'Azur prior to conducting the research. When establishing the initial contact, the purpose of the study was explained to the participants, including what would happen if they agreed to participate, the potential risks and benefits of participating (e.g., revisiting unpleasant memories or contributing to the improvement of future anti-doping programs for cyclists), and ethical considerations. Participants were informed that they had the right to withdraw their consent and discontinue their participation in the study at any time and for any reason, without any repercussions. Confidentiality was ensured, and participants were free to contact us again if they decided to participate. Participants were informed that the interview would take place via video conference and that it would be audio recorded to facilitate data processing. Once they agreed to participate, they were sent a consent form via email prior to the start of the interview. All participants signed informed consent before participating in the study.

### Data collection and processing

The interviews were held between September 2020 and November 2022 via video calls. The interview guide was not modified throughout the data collection phase. However, the initial method for recruiting participants was discussed and revised to immediately provide them with more information concerning data security and the potential implications of their participation.

In preparation for the interviews, the authors (VF and HB) collected information regarding the past activities of each participant through enquiries, searches on sport and anti-doping agency websites, newspapers or books. The information collected allowed the researchers to draw a biographical sketch of the participants’ sporting career, including past performances, selections, results, teams and sometimes doping scandals and penalties. These data supplemented the cyclists’ comments recorded during the semi-directive interviews and helped the researchers to gain an overview as exhaustive as possible of the major events in the participant's life. This methodology is particularly suited to the examination of situations of vulnerability (29, 50).

The interviews were conducted in an online setting, using Zoom software (Zoom Video Communications, 2020), and were audio-recorded on a professional Dictaphone. The researcher conducting the interviews was systematically alone in the room for the entire duration of each interview. The participants could choose the location of their interview. However, it was strongly recommended that they be alone, to ensure that no third party could influence their verbalizations. They were also advised to be in a quiet location, free from distractions, and with a stable internet connection. Despite some minor issues with connectivity loss, participants largely followed these guidelines. This online setting allowed us to extend the recruitment to geographically dispersed and physically distant respondents from across the Atlantic, saving time and money, and allowing a flexible schedule without any problems of jetlag. It also allowed us to continue the research project despite the Covid-19 pandemic. It has been shown that virtual qualitative interviews have the advantage of reinforcing perceived and actual autonomy, in particular when the topic is sensitive, as it is in the case of doping (51–53).

The interview guide was developed with the purpose of addressing the research questions. The guide consisted of a structured set of questions and prompts to ensure consistency and comprehensive coverage of the topics of interest. The interview guide served as a tool for the researcher to ensure that all necessary information was collected from the participants. A pilot interview was conducted with a doping athlete from a different sport (bodybuilding) to check the clarity, relevance, and fluidity of the interview guide. This provided an opportunity to test the guide and make necessary adjustments. Based on the results of the pilot interview, some modifications were made to the guide to ensure that it was effective in addressing the research questions. The pilot interview helped to refine the guide and confirm that it was well-suited to the participants in the study.

The interview guide was divided into four sections. Section one explored the participant's experience as an athlete (e.g., *Can you describe your sports career, from the beginning until now? What are the greatest moments you experienced in your sport?*). The second part aimed to explore individual psychological factors, specifically self-perceptions, motivation to achieve, and the athlete's social context (e.g., *What kind of cyclist were/are you, and how would you describe that athlete? What were your athletic expectations? Could you describe your daily tasks, occupations, and/or hobbies besides cycling during your sports career?*). The third section consisted in describing health behaviors, including eating behaviors, recovery, and performance strategies [e.g., *Can you describe your diet (when you were a cyclist)? What recuperation strategies do/did you use?*]. This third section also allowed us to identify the situations of vulnerability encountered during the athlete's career, and finally to steer the interview toward the issue of doping (e.g., *Could you tell me more about your experience with injuries? Could you describe a situation or an event, when you felt particularly exhausted? What recuperation strategies did you use? Could you discuss the pharmacological preparation you used during your career?*). Finally, the fourth section was optional and was only used when the participant did not spontaneously mention an event identified during the pre-interview investigation (e.g., *Could you describe the context in which this episode took place?*). To conclude the interview, the experimenter was careful to ask the participants how they felt, whether they needed to come back to add something to certain points, and mentioned that psychological support was available upon request.

Individual semi-structured interviews lasted between 52 and 114 min. The audio recordings of the interviews were transcribed *ad verbatim* [e.g., (54)]. Any elements that could lead to the identification of the individual, such as dates, locations, names, or other distinctive features, were not transcribed but were replaced by asterisks to maintain anonymity.

### Data analysis

The thematic analysis followed Braun and Clarke's (55) recommendations, which include: (a) familiarization with the data by the researchers through repeated readings of the interview transcripts to ensure a both broad and deep understanding of the content, (b) generation of initial codes using hybrid inductive and deductive approaches, (c) development of themes to organize and prioritize these initial codes, leading to the identification of overarching themes and sub-themes, (d) review of these themes to ensure the consistency of the code sets within each theme, thereby guaranteeing the coherence of the analytical model, and (e) definition and labelling of these themes. VF, FAL, KC and HB implemented this procedure, and an expert committee comprising all researchers was consulted to improve the precision and completeness of the analysis.

### Quality criteria

Several precautions were taken to ensure the credibility and trustworthiness of the data analysis (56). Participants were given the opportunity to check the transcript and analysis of anonymized interviews, if they wished. Additionally, NVivo coding software (QSR International, 2020; NVivo Version 1.7.1) was used for data analysis, which adds a level of rigor to qualitative research (57). The software allows for precise quantification of the data and of the level of consensus among researchers (58). VF, BH, KC, and FAL read, coded, and compared the coding of the first three interviews (i.e., 30%), first individually and then they discussed them collectively to achieve consensus and ensure that the coding was as homogeneous and objective as possible. The subsequent seven interviews were read and coded, both individually and collectively by VF and BH, and coding was later verified by KC and FAL. After reviewing each new interview, the four authors met to discuss the themes and sub-themes, and to examine the theoretical concepts raised in order to assign categories that satisfied all four of them. This principle of triangulation is known to further enhance credibility by minimizing subjective bias (59). Moreover, regular meetings were organized with the entire group of co-authors to keep them informed and to gather their opinion and feedback on the preliminary results (60).

## Results

Firstly, the substances used by the athletes were identified during the interviews to gain a better understanding of the behavior under investigation. More than fifteen different substances were listed (e.g., erythropoietin “EPO”, amphetamines, testosterone, corticosteroids). Cyclists generally used multiple substances simultaneously, and the products changed throughout their career. EPO was the most prevalent substance used, as shown in this example:

*And from there, we move on to growth hormone, and from growth hormone to testosterone. Because at the time, it was THE cocktail…everyone's cocktail! EPO, growth hormone, testosterone. With a doctor, of course, who oversees it. Because testosterone was measured in milligrams* (P9).

During analysis, codes were defined to represent the situations of vulnerability that contributed to doping. The codes were grouped into four overarching themes: (a) situations of psychological vulnerability, (b) situations of physical vulnerability, (c) situations of relational vulnerability, and (d) situations of contextual vulnerability (see Table 1). The results are presented according to the number of Meaning Units (MUs) and the number of participants. Each final sub-theme is illustrated with one or two verbatim quotes translated into English.

**Table 1:** Themes and subthemes characterizing doping situations of vulnerability.

Themes	Subthemes (b)	Subthemes (c)	Meaning units (participants)
Situations of psychological vulnerability	Negative affects	Emotional exhaustion and cognitive weariness	66 (10)
		Depression	32 (8)
		Stress and anxiety	24 (6)
	Maladaptive motivation	Winning at all costs	82 (10)
		Controlled motivation	22 (7)
		Fear of failure	7 (4)
		Self-sabotage	6 (3)
	Tendency toward moral disengagement		57 (10)
	Reduced sense of accomplishment		29 (7)
	Doping as addiction		23 (4)
	Eating disorders		22 (6)
Situations of physical vulnerability	Physical exhaustion		50 (10)
	Anemia, deficiencies, and perceived need for supplements		31 (8)
	Injuries		3 (1)
Situations of relational vulnerability	Situational temptation of organized doping, social pressure		68 (8)
	Control and psychological or sexual harassment		35 (5)
	Social approval of doping		17 (8)
Situations of contextual vulnerability	Cycling culture		51 (10)
	Environmental and weather conditions		14 (6)
	Competitive stakes		7 (5)

### Situations of psychological vulnerability

The “situations of psychological vulnerability” theme included six sub-themes (MUs = 370): (a) negative affects (MUs = 122), (b) maladaptive motivation (MUs = 117), (c) tendency toward moral disengagement (MUs = 57), (d) reduced sense of accomplishment (MUs = 29), (e) doping as an addiction (MUs = 23), and (f) eating disorders (MUs = 22, see Table 1).

### Negative affects

The “negative affects” sub-theme (MUs = 122) included: (a) emotional exhaustion and cognitive weariness (MUs = 66), (b) depression (MUs = 32), and (c) stress and anxiety (MUs = 24).

### Emotional exhaustion and cognitive weariness

Excerpts from interviews with all ten participants repeatedly alluded to emotional exhaustion and cognitive weariness. One cyclist stated, for example:

*Really, my mind is really, really foggy, I can't…really prioritize things, um, I've become a bit really apathetic, um…So yeah, (doping) is one of the strategies.* (P2)

This mental fatigue eventually reached a point where cyclists began to feel a sense of aversion to their sport. For example, one athlete stated:

*I was tired, same here, but I think it's also emotional and mental fatigue, and you know, I just wanted to do other things. I wanted to go on vacation, by July, I was fed up.* (P5)

This category is therefore the most recurrent situation of psychological vulnerability.

### Depression

Eight out of the ten participants had experienced depressive episodes (MUs = 32) of varying lengths and frequencies, that had direct links to their doping behavior:

*I resisted temptation for a long time, but one day, with temptation all the time, all the time, well, it's hard to resist, and then one day, yeah, precisely in those emotional lows, in those big disappointments, well, we give in. We break, we decide to try it.* (P2)

### Stress and anxiety

Six participants began to dope to cope during phases of anxiety and stress (MUs = 24), as described by these two cyclists:

*Well, there, I doped. I don't know, I'm terrible at managing my stress.* (P6)

And:

*Some were so much more talented than me that I was really terrified of not being up to…to their level. And since it was a team result, you know, I considered myself responsible for the result as the weakest link in the chain. That really stressed me out, you know. (P7)*

### Maladaptive motivation

The “maladaptive motivation” sub-theme (MUs = 117) included: (a) “winning at all costs” (MUs = 82), (b) controlled motivation (MUs = 22), (c) fear of failure (MUs = 7), and (d) self-sabotage (MUs = 6).

### Winning at all costs

The results highlighted a pronounced desire to win that was found in all ten participants, and the number of meaning units for this type of motivation was considerable (MUs = 82). To illustrate the strive for success and for being the best, these cyclists stated:

*I always tried to have a high level in everything I did. It must have been my competitive side, I don't know, I liked it. I liked being the best at what I did. (P3)*

The participants set high standards for themselves, stating for example:

*In any case, whether it's during training…if it's training alone, it's, it's about fighting…it's about competing against myself and against my previous performances.* (P6)

### Controlled motivation

Seven cyclists reported experiencing controlled motivation at times (MUs = 22), notably due to financial stress that made it necessary to achieve results:

*I kind of lost the pleasure of just participating in professional races because of the financial stress and the pressure to justify in the eyes of my loved ones my continued participation in the sport.* (P2)

And:

*I'm going to bring it back to money. It will bring you success. I think I…no, I don't think, I'm certain, I've lived in poverty so much that I mainly wanted to avoid that and provide a different life for my children.* (P9)

### Fear of failure

Seven cyclists also, but to a lesser extent (MUs = 7), expressed a fear of failure, characterized by the fear of stagnating or worse, regressing, and of falling to the back of the pack, as illustrated by these comments:

*Well, I think most good athletes have this concern, this anxiety, or this fear of not disappointing, um…of not disappointing themselves.* (P2)

### Self-sabotage

Finally, it emerged from three interviews that doping allowed the cyclists to engage in “self-sabotage” (MUs = 6), almost implicitly hoping that a positive test would bring an end to the ordeal they were enduring as cyclists:

*And then after two or three days, I said to myself,* “*Come on, what do you have to lose in the end?*” *I thought to myself, at worst, you might get caught, in a way, well, your year is over, and with [him], he'll stop putting you through what he's made you endure.* (P1)

And:

*I wanted instead to have an accident, not to die, I never wanted to die. I never thought about taking my own life. But I wanted to have an accident that was severe enough to let me get out of the sport and get away from him. But in the end, what happened? I failed an EPO doping test.* (P5)

### Tendency for moral disengagement

A tendency for moral disengagement was very prevalent among these cyclists, with all ten adopting some of its mechanisms (MUs = 57). Several mechanisms of moral disengagement were observed:

(a) The almost systematic use of euphemisms to refer to doping:

*At the time, we didn't call it doping, we called it preparation.* (P3)

(b) Or even the diffusion of responsibilities:

*Because, at least at the time, I don't know if cortisone was allowed or not, but everyone was taking it anyway, that's the first thing. And I started using it, not because it had been recommended to me, but because others were doing it.* (P4)

(c) The shifting of responsibilities (e.g., athletes engage in doping because of pressure from their environment):

*I didn't know what it was. I didn't know if it was some kind of doping product or if it was going to give me vitamins or iron. To tell you the truth, I was trying so hard not to look at what it was, I just did what I was told to do, and that was it.* (P5)

(d) A distortion of consequences, minimizing the harm caused and/or the potential effects of the doping substances used:

*Yeah, well, I used EPO, but it was just, let's say, I didn't…it wasn't multiple times.* (P2)

### Reduced sense of accomplishment

Seven cyclists had experienced a reduced sense of accomplishment at times (MUs = 29), as illustrated by this individual:

*Then you realize you're getting dropped (by the pack) and all that, you know. Your morale sinks even lower, and it just drags you down even more.* (P1)

### Doping as an addiction

Four cyclists (MUs = 23) described how their doping behavior followed a pattern of addiction, and how the dependency made them even more vulnerable to doping, to seeking new substances, and/or to increasing dosages. For example, one athlete stated:

*I mean, first, I had to stop using drugs because it was about drugs, not doping, even though it's related because of those amphetamines and those famous* “*Pots Belges*”*.* (P4)

This addiction was either due to the addictive nature of the substance itself:

*And then, when I went off the rails, in the end, I became a drug addict or a guy who is extremely doped, I was no longer a cyclist.* (P4)

In other cases, it was due to the insatiable need to maintain the enhanced level of performance the substances allow:

*Because doping, it makes you…(silence) psychologically, you become dependent. Psychologically, you become dependent on doping. It means that in your head, you say to yourself,* “*I can't ride a bike anymore just drinking clear water. It's over!*” (P9)

### Eating disorders

Longer or shorter periods of disordered eating were also observed in six participants (MUs = 22) characterized by hyper-control and the pursuit of weight loss for improved power-to-weight ratio:

*Then I came back, it was a light lunch that was never enough for me, and I wasn't eating enough. I went through periods of anorexia, but I think it was because it was the only thing I could control.* (P5)

### Situations of physical vulnerability

The “situations of physical vulnerability” theme was broken down into three sub-themes (MUs = 84): (a) physical exhaustion (MUs = 50), (b) anemia, deficiencies, and perceived need for supplementation (MUs = 31), and (c) injuries (MUs = 3, see Table 1).

### Physical exhaustion

All ten athletes reported having experienced situations of physical exhaustion that led them to engage in doping. This category is therefore the most recurrent situation of physical vulnerability:

*That summer, I was really at the end of my rope. I was overtrained, I couldn't do anything at all. I was a wreck. […] At that point, I was chronically tired.* (P4)

### Anemia, deficiencies, and perceived need for supplementation

Eight out of the ten cyclists encountered periods of anemia and deficiencies that led them to use supplements or engage in doping. In other cases, supplements and doping substances were used in anticipation of deficiencies related to their sport practice (MUs = 31). For example, this cyclist explained:

*The solution to get rid of my anemia was to take EPO*. (P5)

The consumption of dietary supplements was occasional at first and became increasingly significant before gradually escalating into doping behavior, as this cyclist said*:*

*I tried creatine for a while. I think it's a little like snake oil. Branch chain amino acids as well. Well, as dietary supplements, yes, protein. I take omega-3 6-9 without really knowing what the benefits are. I'm quite fond of those little pills* (P6).

Another athlete also reported having taken dietary supplements:

*In astronomical quantities.* (P4)

### Injuries

Finally, only one cyclist reported doping after an injury to return more quickly to the previous level (MUs = 3):

*After the second operation on my back, that's when I started (doping). So, if you're injured during the first half of the season, often, you don't have time to train again to achieve a performance during the season, and in high-level cycling, it's a season without any results, it could be the end. So yeah, for me, the temptation was two, three, four times stronger after big periods of what I described to you.* (P2)

### Situations of relational vulnerability

The “situations of relational vulnerability” theme included three sub-themes (MUs = 120): (a) situational temptation of organized doping and social pressure (MUs = 68), (b) control and sexual or psychological harassment (MUs = 35), and (c) social approval of doping (MUs = 17, see Table 1).

### Situational temptation of organized doping and social pressure

Doping behavior often emerged during situational temptation characterized by a form of supervised organization (*n* = 8, MUs = 68), where external members (i.e., often the team doctor or team manager) were in charge of providing substances and managing dosages. The cyclists complied with and followed the instructions of this figure of authority:

*They told me:* “*You don't have to worry; there will be a doctor with you during the preparatory races for the Tour of X, and he will take care of you.*” *That's how I had my first contact with doping.* (P3)

And:

*In the evening, the team's sports director and the doctor came to see me. And then they said,* “*Oh, you just had a fantastic result, have you ever taken cortisone?*” *No. Why?* “*Listen! The doctor is here; don't worry, it's nothing serious. He will give you a protocol, for five days until the end of the race, and he will provide you with cortisone in tablet form.*” (P9)

### Control and sexual or psychological harassment

During the interviews, five cyclists (MUs = 35), representing half of the sample, reported having experienced periods of control and sexual harassment, particularly by the coach. Some of them turned to doping to cope and stay focused following a traumatic event such as sexual abuse or the loss of a loved one. For example*:*

*Me, I'm completely submissive to him now because, because of the violence I experience daily, then with all the threats that he's going to commit suicide, that he's going to murder me and all that. […] His solution to get rid of my anemia was for me to take EPO. I'm [age] years old. I don't know anything about this, I know it's bad, but I tell myself once again:* “*I don't want him to commit suicide.*” (P5)

### Social approval of doping

The cyclist's entourage, particularly family and teammates, appeared to be supportive of their doping behavior (*n* = 8, MUs = 17). Cyclists reported that either their close relatives remained silent:

*Yes, she (my wife) knew. She knew that we had medical preparations, she saw in the fridge that there was a small bag with ampoules in it. So, but she didn't question it, and we didn't talk about it.* (P3)

Or they were even perceived as being encouraging, for example:

*Well, him (my deceased father) would have wanted me to do it (dope). So, he would have given it to me, he would have said,* “*You have to take some stuff, you know.*” *Of that, I am sure.* (P8)

### Situations of contextual vulnerability

The “situations of contextual vulnerability” theme included three sub-themes (MUs = 72): (a) cycling culture (MUs = 51), (b) environmental and weather conditions (MUs = 14), and (c) competitive stakes (MUs = 7, see Table 1).

### Cycling culture

The cultural context appeared to play a significant role in the incentive to engage in doping behaviors. Ten participants mentioned a link between cycling culture and doping (MUs = 51). Specifically, the cyclists identified the pre-Festina era (i.e., before 1998) as a high-risk period for doping:

*I grew up, you know, at a time when the athletes I followed on the Tour de France and in the big famous cycling races in the 90s, were quite doped, so for me, it was a direct association that, as soon as I was going to be in high-level cycling, it would be something I would do, it wasn't a surprise to me that this temptation would come my way.* (P2)

Or:

*The cycling culture means that, well…I'm not talking about today, but I'm talking…it's been almost 40 years. 40 years ago, it was part of the cycling world. It was commonplace, and it was normal. It was normal (laughs) to try things, to try products. Well…the controls weren't as systematic. There were some, but they weren't as systematic as they are today. Well…in the imagination and in the culture of sports at that time, we didn't hide it much. It still happened behind the church, in the village where the criterium was held. But if we were talking about products that were used in that context, otherwise, it wasn't taboo.* (P7)

The cycling culture seemed intrinsically linked to doping or vice versa. This is exemplified by a young cyclist who recounts:

*I thought that EPO, since it's quite well-known in the world of cycling, I figured that if a lot of people were using it, it means that it must really work well, work effectively for a performance in the world of cycling. So, of course, cycling is a sport where you need oxygen, it provides you with more oxygen and everything, so naturally, it must make a significant difference to help you achieve a performance if a lot of people have used it.* (P2)

### Environmental and weather conditions

Because cycling is practiced outdoors, weather conditions such as battling against cold or heat, appeared to have partly motivated doping behavior in six participants (MUs = 14):

*Because it's a tough sport, because you feel cold, because you feel pain, because there's a whole discourse about pain. This is also where aspirations collide…It's very hard to be cold, it's very hard to sleep in depressing places.* (P4)

### Competitive stakes

For half of the athletes interviewed, doping began in situations where the stakes were high and had repercussions on both the athlete's career and finances (*n* = 5, MUs = 7):

*Then when you are an athlete, you are fragile because when there are no results, you worry. You're worried, first because you're in the spotlight, and second is your contract going to be renewed? There are lots of things that come into play, and then worry sets in. So, you're asking for lots of things.* (P3)

## Discussion

The purpose of this study was to explore and to categorize the different situations of vulnerability that predispose athletes, particularly cyclists, to doping. The findings allowed us to identify typical periods during which athletes’ integrity is compromised, thereby facilitating or accelerating their doping behavior. Four major types of situations of vulnerability were categorized: situations of psychological, physical, relational, and contextual vulnerability, contributing useful information to existing knowledge about the difficulties that cyclists may experience with sports), relational (e.g., coaches), and professional (e.g., poor performance).

### Situations of psychological vulnerability

Previous research has reported that the transition to doping is often characterized by fatigue, distress, and a loss of enthusiasm [e.g., (11, 29, 33)], and our findings confirmed these observations. Athletes reported struggling to maintain focus. They felt not only physically but also mentally exhausted, and this affected their clarity of thought over extended periods and consequently, their performance. Although they generally had high perceptions of their competence, many experienced periods of lower achievement, where they felt that no matter what they did, they were failing. These episodes often followed a string of failures in performance and in achieving their goals, as previously noted in the literature [e.g., (29, 33)]. Doping was then viewed as necessary to achieve their goals.

The athletes in our sample were highly prone to other negative affects, such as episodes of depression, stress and anxiety. We observed that more than half of the athletes had periods of stress and anxiety that led them to doping, as recently highlighted in literature about rugby players (37). Our findings aligned with the work of Dydimus and Backhouse (2020), who framed doping more as an act of desperation or a coping strategy than outright cheating, as is predominantly assumed in the existing literature (61).

To our knowledge, the qualitative exploration of maladaptive motivations of doped cyclists has been missing in the literature. The existing discourse-based investigations often overlook the underlying motivational processes that lead athletes to engage in doping behavior. The role of achievement motivation has been explored to date through quantitative methods, predominantly by studying athletes who were not engaged in doping [e.g., (62–65)] which limits the generalizability of the results to the specific population of doped athletes. Our findings clearly revealed a pronounced emphasis on the competitive streak of all the athletes in our sample. The athletes were consistently driven by a desire to excel and an insatiable thirst for victory, similar to Maillot & Meinadier's description of a “champion” (66). In such circumstances, cyclists were willing to do anything to achieve their goals, which is the “winning at all costs” pattern previously emphasized in the doping context [e.g., (36, 67)]. This desire to be the best might partly correspond to the performance-approach goal defined in the 2 × 2 model of achievement goals (68). The existing literature has consistently highlighted the maladaptive nature of this type of goal in relation to doping [e.g., (62–65)].

The participants also reported a fear of failure, albeit to a lesser extent. Athletes seemed to believe they were not worthy and could not tolerate the idea of being deficient in their sport, suggesting they were driven by a mastery-avoidance goal (68). This goal, which was added last to the 2 × 2 framework of achievement goals due to questions about its relevance in sports (68, 69), seems to have meaning in this context. Our results resonated with those of Daumiller et al. (70), suggesting that a mastery-avoidance goal in high-level sports relates to the athletes’ desire to avoid falling short of their high expectations.

Participants also appeared motivated by external forces, such as pressure from the coach or financial constraints, which even sometimes overshadowed their passion for cycling. From the perspective of the Self-Determination Theory (71), the sports literature has extensively shown that controlled motivational regulations are associated with moral disengagement (21, 72)—as also observed in the present study—and positive attitudes toward doping (73).

Finally, the doping behavior of the participants sometimes led to an addiction, either due to the addictive nature of the substance itself, or to the need to achieve a performance. In certain cases, the doping behavior was a coping mechanism motivated by the desire to self-sabotage one's career by the disclosure of doping, whether premeditated or not, effectively ending the ordeal they were enduring. This coping mechanism was a response to the unbearable situation they were experiencing prior to engaging in doping (11, 29).

These findings align with a recent study by Kesenheimer et al. (74), which explored individuals’ motivations for cycling participation across various levels of athletes. The study identified key traits, such as a tendency toward sadomasochism and sensation-seeking, as primary motivators. Cyclists may therefore inherently possess traits that increase risk, particularly among those driven by a “win-at-all-costs” mentality, who often reach elite levels. In these athletes, vulnerable situations that hinder their goals could trigger doping behavior.

Our findings also corroborated existing literature on the prevalence of disordered eating behaviors among these athletes [see for review (75)] driven by the quest for lower weight, a more optimal power-to-weight ratio, and perhaps most importantly, a need for hyper-control. While not specifically categorized in this study, it is reflected in the way every aspect of their lives is calculated and meticulously planned, from their training regimes and intensity to their equipment and daily schedules. In a recent study, Scoffier-Mériaux et al. (76) demonstrated that healthy eating behaviors mediated the relationship between self-determination motivational constructs and the tendency of elite athletes to engage in doping, suggesting the need for further exploration in this area. Our work confirms the link between disordered eating behaviors and doping, and also advocates for additional research on the subject.

### Situations of physical vulnerability

Due to the inherently exhausting nature of cycling [e.g., (6, 7)], all participants in our sample experienced physical exhaustion. We were surprised that only one participant identified periods of injury as a time of vulnerability to doping. This individual, in line with existing literature on the topic [e.g., (30, 34)], stated that the temptation to dope was irresistible during these periods, when it seemed to be the only solution for rapid recovery in time for seasonal races. Given that literature highlights post-injury recovery periods as high-risk phases for doping, we questioned participants about their history of injuries and the recovery techniques they used. When asked directly about doping, participants unanimously denied engaging in it during the specified periods. They explained that during those times, they distanced themselves from both sports and maladaptive behaviors. Doping typically occurred when they were nearing their physical or mental limits, using it as a way to overcome these barriers. In contrast, athletes recovering from injuries used the time to rest, sleep, and reconnect with loved ones.

A relevant parallel can be made between phases of deficiency and anemia, which led athletes to overconsume dietary supplements, sometimes escalating into doping, as explained by the Gateway Theory [e.g., (77, 78)]. This pattern was evident in the study. While athletes could still train during periods of deficiency or burnout, their performance suffered. In contrast, fatigue or visible injuries (e.g., fractures) prevented them from practicing at their usual level, highlighting a key difference that may have influenced their doping behavior.

When considering situations of psychological and physical vulnerability collectively, our findings revealed that athletes were most vulnerable to doping in situations characterized by physical, emotional, and cognitive exhaustion, along with a diminished sense of accomplishment. These conditions often culminated when associated with negative feelings toward their sport and even disgust for it. Athletes pushed themselves into extreme states of exhaustion and maintained those states, ultimately driving themselves into situations where doping seemed to be the only viable solution [e.g., (11, 12, 33)]. These various categories are in line with the dimensions of athlete burnout, as defined by the most recent understandings of the syndrome [e.g., (79, 80)], which is well-known in the sports environment. Burnout leads to various negative outcomes for athletes, including quitting the sport [e.g., (81)], sleep disturbance (82), and compromised immune functions, among others (83). However, to our knowledge, no study to date has identified the role of burnout in relation to doping in sports.

### Situations of relational vulnerability

The present qualitative study examined doping behaviors in cyclists and offered insights into unique aspects of the situations of relational vulnerability they encounter. Interestingly, our findings show that doping sometimes appeared in a context where temptation was induced by a medical professional or team supervisor, who promised success in exchange for trust and cooperation in doping. However, the same athletes described instances where doping occurred in situations of solitude, without prior discussion or consultation with anyone. They turned to doping when they felt they could no longer meet the challenges before them and failed to seek the necessary help. These findings therefore suggest that relational vulnerabilities change over the course of an athlete's career. These observations align with the existing literature, which suggests that doping-related actions sometimes appear within a set of coordinated and collective actions among a group of athletes, managed in a hierarchical manner [e.g., (25, 26, 33)]. In other cases, doped cyclists appeared immune to any external environmental influence unrelated to training and performance (29, 33). Some even began using performance-enhancing substances without having explored other options (11).

An unexpected finding, at least in terms of its significance, was the prevalence of psychological and sexual harassment and abuse within the cycling community. This issue was spontaneously mentioned by half of our sample, consisting of both male and female participants. Doping then occurred either (a) because the coach explicitly imposed it, (b) because the athletes considered doping as the only way to live up to the expectations of their tormentor, (c) or even to express emotions which were unbearable. The phenomenon could be attributed to the influential role of coaches (84) and sports directors within the cycling environment (85). Moreover, the organizational structure of cycling (86), in which athletes depend on team dynamics and results (14) should be investigated in greater depth. Certain personality traits of high-level athletes, particularly cyclists (74), such as high levels of perfectionism, could be a factor that makes them more vulnerable to the influence of a figure of authority promising success (i.e., the coach). This category points to additional systemic issues that may not only influence doping behavior but also create a culture of vulnerability and exploitation that needs to be considered.

Furthermore, cycling is a working-class sport, deeply rooted in the family sphere (87). Cyclists reported that spouses or parents who were aware of their doping habits often displayed no strong reaction. None of the athletes reported experiencing violent responses or outright rejection from their families; however, some did mention the concerns of relatives about the potential health risks associated with substance use. One cyclist expressed belief that his deceased father, who was also a cyclist, would have endorsed his decision to take performance-enhancing substances, viewing it as a necessary part of “doing the job”. Therefore, the family-oriented nature of cycling culture added another layer to our understanding of doping behavior in this sport. It confirmed the interplay of social norms and expectations that might contribute to initial or continued doping [e.g., (88)]. It could also imply that the acceptance by close family members, or at least the lack of overt disapproval may serve to further normalize doping behavior, reinforcing the moral disengagement mechanisms identified.

### Situations of contextual vulnerability

As already mentioned, we observed that doping in cycling occurred both in organized and structured behavior and in isolated acts “away from prying eyes.” Our results confirm a historical legacy of organized doping, often within the confines of a team setting [e.g., (12, 25)]. However, first encounters with doping occurred either in a supervised manner or autonomously. A smaller subset of athletes, mainly from younger generations, initiated doping on their own, without discussing it within their sports environment. This shift reflects a changing cultural context, notably influenced by the Festina scandal, which has left a lasting impression as evidenced by our interviews. The fact that doping appears to be increasingly perceived as a solitary act is consistent with the existing literature on the topic [e.g., (89)]. However, the belief persists among these young, independently doping athletes that “everyone is doing it”. This diffusion of responsibilities is a mechanism inherent to moral disengagement, as discussed above [e.g., (21, 22)].

Another contextual feature intrinsic to cycling is related to the physical demands of a sport that takes place outdoors. More than half of the sample highlighted the challenges presented by environmental conditions. Struggling for hours against cold weather or maintaining a prolonged effort in scorching heat and under a blazing sun appeared to contribute to the development of a doping behavior among these athletes. The demands in terms of training and performance, combined with weather conditions, can influence the overall health of the athlete during periods of intense training or performance (90).

Finally, the influence of the competitive stakes was observed during the interviews but to a lesser extent. This observation moves us away from the simplistic view that doping is solely a matter of money, and instead highlights the complexity of its underlying causes (91). This particular rationale was notably absent from the list of reasons for doping compiled by Bilard et al. (13).

### Limitations and perspectives

Despite the many insights provided by this study, a few limitations must be discussed. Given the prohibited and socially unacceptable nature of doping in sports (92, 93), it was challenging to gain access to doped athletes [e.g., (50)]. This made it necessary for us to expand our sample to include athletes from other French-speaking countries in order to achieve data saturation. The broadening of inclusion criteria resulted in a heterogeneous sample, particularly in terms of gender, age, and nationality. Our sample was characterized by a low representation of women, reflecting the male-dominated nature of cycling. Since doping behaviors vary by gender [e.g., (23)], future studies will need to be conducted to complement our results. The athletes interviewed were elite-level cyclists, though not all professional, and we know that they have all participated in the same races, circuits, and teams, and have attended the same team-building camps, albeit at different times. However, we must acknowledge that our findings remain descriptive and are too limited in scope to determine how these sociodemographic characteristics influenced the results. Additionally, our sample might have suffered from selection bias (94), given that the volunteers may have been athletes who have recovered particularly well from their doping experiences or who exhibit a strong sense of redemption. It would therefore be worthwhile to further investigate the impact of these differences on the outcomes, and to do so on a larger scale whenever feasible.

Our qualitative study was successful in following the athletes’ journey and identifying challenging situations encountered during their career that led them to doping. However, the existing analytical framework falls short in investigating the interplay between the different features observed. Alternative methodologies, such as interactionist approaches or life-course analyses, could fill this gap [e.g., (11, 33)]. Replicating the study using these methodologies and expanding the analytical focus to include motivational factors and situations of vulnerability are intriguing avenues for future research. Moreover, follow-up interviews with the participants in our qualitative investigation could have allowed for re-enactment, thus providing a more thorough and accurate account of the events (29). We urge researchers conducting future qualitative studies involving doped athletes to consider these methodological opportunities.

Furthermore, we acknowledge that some situations of vulnerability were assigned to one category, when they could have belonged to, or overlapped with, multiple categories simultaneously. For instance, physical exhaustion is closely linked to mental exhaustion, and some of the participants themselves emphasized the connection. This categorization is thus a proposal that warrants further discussion.

Lastly, the interviews, conducted across different continents and during the COVID-19 pandemic, used video conferencing due to restrictions. While this method allowed data collection, it introduced limitations such as a loss of intimacy, potentially affecting the holistic quality of qualitative research (95). To address these issues, specific efforts should be considered, and future research should aim to conduct in-person interviews to minimize biases associated with virtual communication.

## Conclusion

This study allowed us to expand our understanding of the situations of vulnerability that predispose cyclists to doping, and to provide a clearer categorization of these situations. This comprehensive view offers a holistic understanding of the various situations of vulnerability that converge toward doping, and include physical, psychological, relational, and contextual aspects.

We observed that situations of psychological vulnerability such as negative affects make the cyclist's performance goals unattainable without the external aid of doping. Doping was found to be a strategy to cope with these challenges. It not only fulfills their thirst for victory but also sometimes serves as an emotional escape or a last resort when the pressure becomes unbearable. Doping in cycling has its own unique context, colored by a heavy history in which doping was once organized and structured within the team framework. The demanding nature of the sport pushes cyclists to adopt strategies that may skirt the rules, to address issues such as inadequate recovery, financial pressure, harassment, or challenging weather conditions. Doped athletes, deeply characterized by their overwhelming desire to be champions and to be the best, are at greater risk precisely because they seem unstoppable, even in the face of exhaustion or repeated failure. We can hypothesize that those less driven by a thirst for victory, perhaps less resilient, may be more conscious of their limits and recognize their need for recovery and may even be able to reconsider their high-level career ambitions. This analytical framework should pave the way for future research in related vulnerabilities, alongside dispositional factors. Practically, it should also contribute to better screening and prevention of doping, and provide a more favorable environment for athletes.

## Data Availability

The raw data supporting the conclusions of this article will be made available by the authors, without undue reservation.
